# A Journey With Allergy: A Recollection

**DOI:** 10.1097/WOX.0b013e31821f3f67

**Published:** 2011-05-15

**Authors:** Felicidad Cua-Lim

## 

Memories can be the most precious of possessions. Today I write about over 50 years of my journey with allergy. More than naming my field of specialization, allergy has been my passion, and has become, in its way, my own life's companion.

My journey of love with allergy began in 1960 at the Royal Victoria Hospital in Montreal, Canada. I believe I was the first Asian to have trained under the legendary Dr. Bram Rose, a brilliant disciplinarian who was then President of the American Academy of Allergy (now the American Academy of Allergy, Asthma, and Immunology, or AAAAI). My husband, Dr. Manuel Lim, had begun his training in Otolaryngology at the Royal Vic under Dr. James McNally the year before. Manuel had made an appointment for me with Dr. Rose that July. I will never forget my first meeting with Dr. Rose in his office. His directives to me, a young fellow about to embark on a career in allergy, were blunt, to say the least: "When you do not know the answer, say that you do not know it; and when you are not sure, say nothing. I cannot stand people who talk through their______." His colorful words are forever carved in my memory, because they amounted to a rigorous standard for working in our specialty: be accurate, tolerate no nonsense. In the decades to come, I learned to fully appreciate my training under Bram (as I finally came to call him, decades later, in 1991) (Figure 1). He absorbed the gist of information like a sponge, synthesizing the essentials. At conference discussions, he handled controversies skillfully and artfully, never losing his cool.

**Figure 1:**
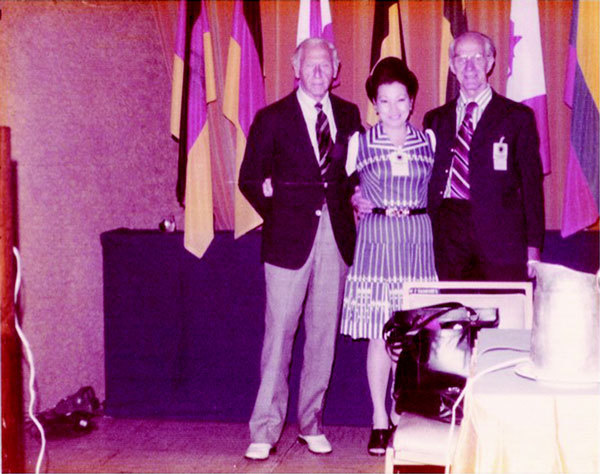
**From left to right: Bram Rose, Felicidad Cua-Lim, and William Frankland at an IAACI meeting in the 1980s**.

In 1960, after a month of study under Dr. Art Leznoff, I worked with Dr. Herbert Blumer for the next 2 years. Herbie was very important in my pursuit of learning, as were the consultants who guided me through the rest of my training at Royal Vic: Dr. Vevvy Leith and Dr. Allan Knight. Instrumental to my Master of Science degree was Dr. Max Richter, who taught me to love research. Inquisitive, probing, and ingenious, Max represented a different approach to allergy training than Bram, and I benefited from both mentors immensely. Another impressive personality was Dr. Alec Sehon, who liked to call me "tahimik, " a Filipino word meaning "quiet." To all my colleagues at Royal Vic and McGill University in 1960 to 1963, Fellows, technicians, and nurses (especially Kay Thomas), I owe what I am today. They form the pillars of my professional life.

I was allowed to see patients after a month at Royal Vic, and after 3 months I became part of the team, presenting at journal clubs and joining research. In September 1960, 6 months after my arrival at Royal Vic, Bram sent me with the "boys" to Washington, DC, to attend my first American Academy of Allergy meeting at the height of winter. A generous mentor, Bram introduced me to luminaries of allergy in the 1960s (Dr. Harry Alexander, Dr. Dan Campbell, Dr. Bill Sherman, Dr. Francis Lowell, Dr. Sam Feinberg, Dr. Max Samter, among others), and to younger physicians who would become famous in the years to come (Dr. Phil Norman, Dr. Sheldon Siegel, Dr. Frank Dixon, to name only a few). This early mentorship and collegiality sparked an intense desire for the pursuit of knowledge in allergy, a solid foundation for what I could become as an allergist.

In 1961, Bram gave me a grant to attend my first International Association of Allergology and Clinical Immunology (IAACI) convention. It was a thrilling and terrifying experience for me, because all the "giants" in the field (the likes of Dr. Bernard Halpern and Dr. Jimenez-Diaz) were in attendance at New York City. In fact, the IAACI, now the World Allergy Organization (WAO) has become a tradition for me: I have not missed a meeting since 1967, the year when I also became Fellow of the American Academy of Allergy. A year after I qualified for the American Board of Allergy and Immunology in 1975, Dr. Alec Sehon nominated me to serve as member-at-large of the IAACI Executive Board a year later. It was a great privilege for me to be the first female physician to sit on the IAACI board.

Let me now switch gears and describe the early decades of our specialty in the Philippines. I returned to my homeland from my training in Royal Vic in June 1963, and my arrival caused quite a stir. At that time, allergy was still considered a "controversial subspecialty." Asthmatics were in the hands of pulmonologists, patients with atopic eczema/dermatitis and urticaria were being seen by dermatologists, and those suffering from allergic rhinitis were treated by Otolaryngologists. In such a situation, my friends in Manila worried about my potential patient base as an allergist. In fact, at one of my first public lectures on allergy, our specialty was dismissed by a prominent Philippine University's Chair of Dermatology who stated that "allergy is a waste basket diagnosis." I was crushed, but Dr. Arturo B. Rotor, my country's first trained allergist and Assistant Dean of the University of the Philippines' College of Medicine, defended my presentation as a concise summary of the state of the field. Encouraged, I began consulting with Dr. Rotor about my idea of organizing a national society of allergy, but at that point, such a dream seemed unrealistic since there were only 5 allergists in the country.

The discovery of IgE by Professors Bennich and SGO Johannson (Figure 2) and Kimishigi and Teruko Ishizaka (Figure 3), officially accepted by the World Health Organization in 1967, changed everything. A rigorous understanding of the antibody culprit behind allergic reactions transformed the science and practice of allergy, lifting allergists above the taint of quackery in the Philippines. In 1971, Dr. Rotor called me to tell me that the time had come: I set about organizing a national professional organization of allergists. I was elected the founding President of the Philippine Society of Allergology and Immunology (PSAI) in 1972, with an initial membership of about 10 physicians.

**Figure 2:**
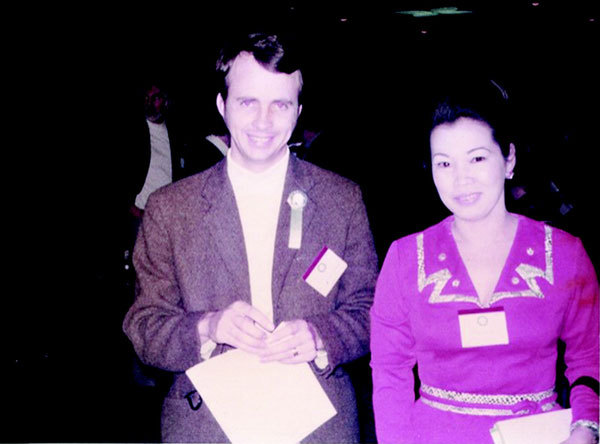
**SGO Johansson and Felicidad Cua-Lim at an IAACI meeting in the 1970s**.

**Figure 3:**
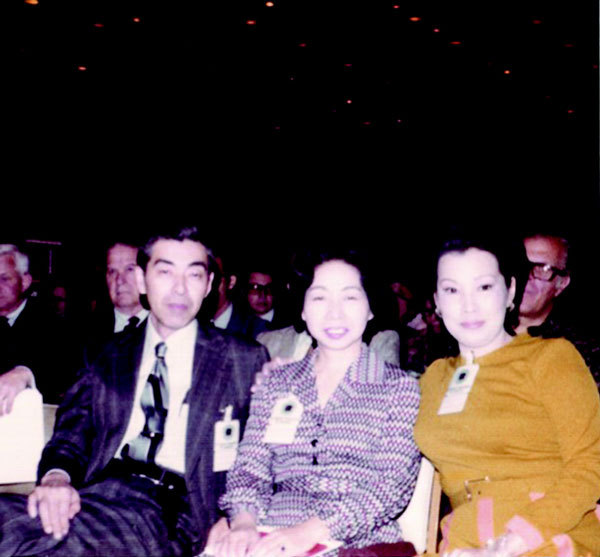
**From left to right: Kimishigi Ishizaka, Teruko Ishizaka and Felicidad Cua-Lim at an IAACI meeting in the 1970s**.

The 1970s were a formative period for allergy in the Philippines. In the early 1970s, with the help of palynologist Dr. Pacifico Payawal and pharmacist Gloria Laserna, both formerly with the National Science Development Board (NSDB), I conducted pioneering pollen surveys in 5 regions of the Philippines and mold surveys in Metro Manila. I conducted these studies because, as Oren Durham once said, "the allergist must know his pollens." Morris Webb of Hollister-Stier laboratories and Dr. Bob Esch of Greer laboratories created extracts from the local pollen fiora I supplied, while with the help of Dr. Vin Bristow, the Commonwealth laboratories of Australia, Dr. John Trinca, and Kathy Stringer, we were able to analyze and identify Philippine house dust mites. Three pioneering Filipino allergists (Dr. Benigno Agbayani, Dr. Miguel Noche, and myself) disseminated the knowledge of allergy and immunology to urban centers and rural towns in the Philippines, using chalkboards and mimeographed handouts, speaking to both friendly and pessimistic audiences of various religious and ideological groups. A handful of pioneering researchers on allergy represented various Philippine universities: myself and Dr. Noche of the University of Santo Tomas (UST), Dr. Agbayani and Dr. A. Lingao of the University of the Philippines (UP), and Dr. Manuel Ferreria of Manila Central University. I also remember Dr. Avelina Bacnis for her principles and discretion. A Secretary of PSAI for more than ten years, Dr. Bacnis refused to be elected President. And I appreciate the contributions of another of the ten founding members of PSAI, Dr. Amelia Ochoa-Bulmer, who served as Secretary-Treasurer of PSAI and who is currently in private practice in the Philippines. At present, research is being carried on by a younger generation of Filipino allergists, among them, Dr. Agnes Gonzales-Andaya and Dr. Remedios Ong at UST, Dr. Madeleine Sumpaico at UP-Philippine General Hospital, Dr. Manuel Po and Dr. F. Padua at Fe del Mundo Medical Center and National Kidney and Transplant Institute. The PSAI became a member of the IAACI in 1976 and today, what is now called the Philippine Society of Allergy, Asthma, and Immunology (PSAAI) has nearly a hundred members. The PSAAI is in very good hands.

One of PSAAIs greatest accomplishments, to my mind, is our hosting of the 1998 Third Asia Pacific Congress of Allergology and Clinical Immunology in Manila. Graced by the presence of then WAO president, Prof. SGO Johannson, and over 30 international speakers, the Congress drew a record number of attendees, with over 1300 participants from all over the world. I remain incredibly grateful to WAO for honoring me in Munich, Germany with the 2005 Outstanding Clinician Award; this was the greatest surprise of my life, and to this day I feel humbled, honored, and privileged to be so recognized. In coming to the final section of this editorial (my debut as an editorial-writer), I would like to thank Professor Lanny Rosenwasser and Sofia Dorsano for their invitation to contribute my perspective on changes in our field and the continuing importance of global exchanges to our specialty. In that spirit, I would like to offer these observations and recommendations:

I would like to see the WAO continue to play a formative part in encouraging the development of national allergy societies throughout the world. For nations in what used to be called the "Third World" and is today referred to as the "Global South, " honoraria for the most distinguished and sought-after international speakers can be prohibitive. WAO's help in this regard--either through cosponsorship of speakers or organizing volunteer lecturers to speak at national allergy societies overseas--could make a crucial difference. WAO, through the World Allergy Forum and (GLORIA), has already been giving grants to national allergy societies of at least one speaker for their conventions, and I would like to see such grants expanded. A tandem between a clinical allergist and a clinical immunologist would be ideal. Locally, I believe that continuing medical education credit requirements should be mandatory for the national medical societies.

In an era of transnational economic recession, many allergists who would like to attend international conventions may be barred from doing so by financial considerations. Discounts could be given to delegates from developing nations and perhaps, like the AAAAI and ACAAI, seniors (who, like me, still have much to contribute) could be offered free or discounted registration. In the Philippines today, both government grants and pharmaceutical sponsors rarely underwrite the travel costs of delegates to international medical conventions; in the few occasions when they do, they tend to prioritize infectious diseases and cardiovascular and endocrine disorders to the detriment of immunology and allergy.

Dr. Allen Kaplan and Dr. Carlos Baena-Cagnani, both Past Presidents of WAO, were among the first to voice the ideal of a global exchange of ideas on allergy, allowing poor and wealthy nations alike to share ideas. In my view, national allergy societies play a crucial role in both national and global exchanges, providing critical education and training for specialists and allowing collegial partnerships and discussions with other specialists. By eludicating proper management techniques, such national and transnational fora ultimately help to improve the quality of life for our patients.

## End note

Felicidad Cua-Lim, MD (Magna cum Laude, Meritissimus, University of Santo Tomas, 1955; MSc, McGill University, 1962) is the Founding President of the Philippine Society of Allergology and Immunology (1972-1978) and has served as Honorary President of the Philippine Society of Allergy, Asthma, and Immunology from 2000 to the present. She was also Founding Chair of the National Asthma Movement (1994-1996), President of the Philippine College of Physicians (1996-1997), and President of the Asia Pacific Association of Allergology and Clinical Immunology (1998-2000). In addition to being named Outstanding Clinician by the WAO in 2005, she was honored as Outstanding Physician of the Year by the Professional Regulation Commission in 1984, Honorary International Distinguished Fellow by the American College of Allergy and Immunology in 1990, and Most Distinguished Physician by the Philippine College of Physicians in 2002. She taught at the University of Santo Tomas for 40 years, retiring as Professor 1 in 1995.

